# Effects of adrenergic-stimulated lipolysis and cytokine production on in vitro mouse adipose tissue–islet interactions

**DOI:** 10.1038/s41598-022-18262-0

**Published:** 2022-09-22

**Authors:** Morgana Barroso Oquendo, Estela Lorza-Gil, David Juarez-Lopez, Robert Wagner, Andreas L. Birkenfeld, Susanne Ullrich, Felicia Gerst

**Affiliations:** 1 Division of Endocrinology, Diabetology and Nephrology, Department of Internal Medicine IV, University Hospital Tübingen, Tübingen, Germany; 2 German Center for Diabetes Research (DZD E.V.) and Institute for Diabetes Research and Metabolic Diseases of the Helmholtz Center Munich at the Eberhard-Karls-University of Tübingen, Neuherberg, Germany

**Keywords:** Endocrinology, Endocrine system and metabolic diseases, Diabetes, Obesity

## Abstract

Inflammatory cytokines and non-esterified fatty acids (NEFAs) are obesity-linked factors that disturb insulin secretion. The aim of this study was to investigate whether pancreatic adipose tissue (pWAT) is able to generate a NEFA/cytokine overload within the pancreatic environment and as consequence to impact on insulin secretion. Pancreatic fat is a minor fat depot, therefore we used high-fat diet (HFD) feeding to induce pancreatic steatosis in mice. Relative *Adipoq* and *Lep* mRNA levels were higher in pWAT of HFD compared to chow diet mice. Regardless of HFD, *Adipoq* and *Lep* mRNA levels of pWAT were at least 10-times lower than those of epididymal fat (eWAT). Lipolysis stimulating receptors *Adrb3* and *Npr1* were expressed in pWAT and eWAT, and HFD reduced their expression in eWAT only. In accordance, HFD impaired lipolysis in eWAT but not in pWAT. Despite expression of *Npr* mRNA, lipolysis was stimulated solely by the adrenergic agonists, isoproterenol and adrenaline. Short term co-incubation of islets with CD/HFD pWAT did not alter insulin secretion. In the presence of CD/HFD eWAT, glucose stimulated insulin secretion only upon isoproterenol-induced lipolysis, i.e. in the presence of elevated NEFA. Isoproterenol augmented *Il1b and Il6* mRNA levels both in pWAT and eWAT. These results suggest that an increased sympathetic activity enhances NEFA and cytokine load of the adipose microenvironment, including that of pancreatic fat, and by doing so it may alter beta-cell function.

## Introduction

Obesity and visceral fat accumulation are major risk factors for the development of type 2 diabetes (T2D)^1^. Adipose tissue is a metabolically active tissue and diet-induced obesity links to accumulation of visceral fat with negative impact on glucose homeostasis^2^. Convincing evidence indicates that persistent hyperglycaemia develops when pancreatic beta-cells fail to secrete sufficient insulin to compensate for insulin resistance^3^. The role of pancreatic fat for beta-cell function is controversially discussed^4^. Association studies using unstratified human cohorts found no correlation between pancreatic steatosis and reduced insulin secretion^5^. On the other hand, we described that increased pancreatic fat correlates with lower insulin secretion in subjects at high genetic risk for T2D and in individuals with impaired fasting glucose (IFG) and/or impaired glucose tolerance (IGT)^6,7^. In accordance, a recent work reported that removal of peripancreatic fat in HFD-fed mice correlated with increased islet expression of markers of glucose-stimulated insulin secretion^8^. These findings suggest that pancreatic steatosis may aggravate or accelerate the development of islet dysfunction and consequently promote hyperglycaemia. Pancreatic fat is a tiny fat depot in comparison to subcutaneous and visceral fat compartments^8^. Therefore, we hypothesize that pancreatic fat exerts rather local, paracrine effects on islet function instead of inducing systemic changes^9^. Metabolic factors secreted from adipocytes and known to impair function and survival of beta-cells are cytokines and saturated fatty acids^10–12^. Of note, unsaturated fatty acids support GSIS and survival of beta cells^10^. A recent work showed that fatty acids released from pancreatic adipocytes may cause insulin hypersecretion in islets isolated from a diabetes prone mouse model^13^. A persistent insulin hypersecretion may exhaust beta-cells and render them dysfunctional. Previously, we reported that lipolytic activity of in vitro differentiated human primary pancreatic adipocytes depends on donor’s metabolic status, i.e. it is lower in adipocytes of subjects with T2D than of those with normal glucose tolerance (NGT)^14^.

Obesity and, in particular, ectopic fat accumulation are drivers of low-grade inflammation. The percentage of adipose tissue macrophages increases from 5% in lean up to 50% in obese rodents and humans^15^. Beside increased immune cell infiltration, macrophage proliferation was detected in obese adipose tissue^16,17^. An increased production of inflammatory cytokines, in particular IL-1β, impairs beta-cell function and survival^9,18,19^. The simultaneous exposure of beta-cells to increased levels of NEFA and of inflammatory cytokines activates divergent stress pathways and impairs insulin secretion, thereby contributing to the hyperglycaemic episodes^20,21^. Finally, hyperglycaemia and NEFA may further accelerate inflammation via TLR4 activation and IL-1beta production^22,23^.

In order to evaluate the impact of pancreatic adipocytes on islet function, we assessed the lipolytic (NEFA release) and secretory (cytokines and adipokines expression) potential of pWAT and compared it with that of epididymal (eWAT) and subcutaneous, inguinal (iWAT) fat of chow (CD) or high -fat diet (HFD) fed mice. HFD was used to induce an obese environment, in particular pancreatic steatosis and insulin resistance in C57BL/6 mice. To investigate the adipocyte-islet interactions, the fat pads and islets were subjected to short-term co-incubation assays in the presence of substances relevant for insulin secretion and lipolysis.

## Materials and methods

All experiements performed in this study are reported in accordance with the ARRIVE guidelines.

### Animal handling and diets

C57BL/6 N mice at the age of 4 weeks were randomly assigned to two groups. Animals of the control group were fed a standard chow diet (CD, 10 kcal% fat), while the test group was fed with a high-fat diet (HFD, 45 kcal% fat, #D12451, Research Diets, New Brunswick, NJ, USA) for 20 ± 1.1 weeks. The animal experiments were approved by the local responsible authorities (Approval #M10-18G from 18. 09. 2018 of the Regierungspräsidium Tübingen, Germany). Animal care and handling was conducted in compliance with the German animal protection law and the Directive 2010/63/EU of the European Parliament on the guidelines for the protection of animals used for scientific purposes.

### Assessment of blood parameters and tissue processing

Fed blood samples of a representative number of animals (*n* = 11/group) were collected in order to assess the in vivo diet-induced NEFA load. There were no sex-associated differences, since blood values were similar. Blood glucose was measured using a glucose analyzer (Accu-chek® Performa, Roche Diagnostics, Rotkreuz, Switzerland). Serum samples were frozen for measurements of insulin, triglyceride, and NEFA concentrations using commercial kits from Mercodia (Uppsala, Sweden), Siemens Healthcare Diagnostics (Advia® Chemistry, Erlangen, Germany), and Sigma-Aldrich (Munich, Germany), respectively. Serum insulin was measured in 7 and 8 mice fed CD or HFD, respectively. Adipose tissues were snap-frozen for RNA isolation or immediately processed for functional measurements as described below.

### Quantitative RT-PCR

Fat pads were minced in RNA extraction buffer and homogenized in a TissueLyser II (Qiagen, Hilden, Germany). The homogenates were then centrifuged at 12,000 g, for 20 min at 4 °C. The lipid layer was discarded and the aqueous phase collected for RNA purification.

Total RNA was isolated (NucleoSpin® RNA and NucleoSpin® RNA XS, Macherey–Nagel, Düren, Germany) and RNA integrity evaluated (Bioanalyzer 2100, Agilent Technologies, Santa Clara, CA, USA). cDNA was synthetized from RNA samples with RIN > 6 using random primers (Transcriptor First Strand kit, Roche Diagnostics). Semi-quantitative PCR was performed with the LightCycler 480 system (Roche Diagnostics) using specific primers (Invitrogen, Carlsbad, CA, USA) and probes (Roche Diagnostics). All primers and probes are given in Suppl. Table 1. Normalized gene expression was calculated as the ratio of C_t_ of target transcripts vs *Rps13*, used as housekeeping gene (2^−ΔCt^_,_ ΔC_t_).

### Lipolysis assay

Lipolysis was performed with freshly collected WAT depots as described by Baskaran and Thyagarajan^24^. In brief, fat pads were washed with PBS at room temperature and cut into small pieces (< 5 mg). Inguinal (iWAT, total 20 mg/0.2 ml), epididymal (eWAT, 20 mg/0.2 ml) and pancreatic (pWAT, 10 mg/0.2 ml) fat pads were preincubated and then incubated, each time for 60 min at 37 °C and 5% CO_2_-humidified atmosphere, in DMEM containing 4.5 g/l glucose and supplemented with 2% fatty acid-free BSA and 5 µM triacsin C. Isoproterenol, adrenaline or atrial natriuretic peptide (ANP) were added during preincubation and incubation as indicated in each experiment. Thereafter, NEFA and glycerol release was assessed in the incubation supernatant using commercial reagents and kits (Sigma-Aldrich). The amount of released NEFA and glycerol was normalized to protein content. Proteins were extracted by lysing the fat pads in PBS supplemented with 0.1% Triton-X-100 followed by centrifugation at 12,000 g for 20 min at 4 °C. Protein concentration was measured in the supernatant using Bradford assay (BioRad Laboratories, Munich, Germany). Isoproterenol and adrenaline bitartrate salt solid were purchased from Sigma-Aldrich. Triacsin C and murine atrial natriuretic factor (1–28) trifluoroacetate salt (ANP) were purchased from Tocris Bioscience (Bristol, United Kingdom) and Bachem (Bubendorf, Switzerland), respectively.

### Islet isolation

All animals were euthanasiated (CO_2_ inhalation) before the islets were isolated, in accordance with the animal experiments approval. Collagenase solution (1 mg/ml collagenase #NB8, Serva) was injected into the ductus choledochus, the pancreas removed and digested for 10 min at 37 °C. Islets were separated from the exocrine tissue under the dissection microscope using ice-cold Hank’s balanced salt solution supplemented with 0.5% (wt/v) BSA as described previously^25^. Islets were cultured overnight in RPMI1640 medium (Lonza) containing 2 g/l glucose and supplemented with 10% (v/v) FCS, 10 mM HEPES, 2 mM L-glutamine, and 1 mM Na-pyruvate.

### Co-incubation of murine islets and fat pads

The fat pads were minced and pieces of eWAT (≤ 20 mg/well) and pWAT (≤ 10 mg/well) were distributed in a 48-well plate and preincubated (1 h at 37 °C) in KRB containing 2.6 mM CaCl_2_, 5.6 mM glucose and 1% (wt/v) fatty acid-free BSA. Thereafter, fat pads were transferred to fresh wells containing 0.25 ml KRB and test substances as indicated in each experiment and incubated for 1 h. The overnight cultured islets were preincubated (1 h at 37 °C) in KRB containing 2.8 mM glucose and 0.5% (wt/v) BSA. Thereafter, batches of 5 islets/condition were added to the fat pads-containing wells. The co-incubation was conducted for an additional 1 h in KRB containing 0.5% fatty acid-free BSA and test substances at the appropriate concentrations. Secreted NEFA, secreted insulin and islet insulin content extracted with acid ethanol were measured using ELISA kits (Sigma and Mercodia, respectively).

### Histochemistry of mouse pancreatic adipose tissue

Whole pancreatic adipose tissue pads were used for histochemical analysis. Formalin-fixed paraffin-embedded tissue blocks were sectioned (3–6 µm) and tissue sections were treated with xylene, descending ethanol dilution series, and distilled water to dewax the tissue samples. Thereafter, sections were stained with hematoxylin/eosin solution and visualized using a Zeiss light microscope. Randomly selected images were taken at 20 × magnification.

### Statistics

Data are presented as means ± SEM for the given number of replicates. Statistical analysis was performed using GraphPad Prism (Version 9.1.2, GraphPad Software Inc., La Jolla, CA, USA). Differences between two groups were assessed by Student’s t-test. ANOVA with Tukey post-testing was used when more than two groups were compared. Differences were considered statistically significant at *p* ≤ 0.05. The ROUT method (coefficient Q 5%) was used to detect and exclude outlier data.

### Human pancreatic fat cell analyses

The study protocol for the characterization of human pancreatic fat pads and the differentiation and characterization of adipocytes were approved by the Ethics Commission of the Medical Faculty and the University Hospital of the University of Tübingen in accordance with the Declaration of Helsinki (697/2011BO1 and 563/2019BO2). Written informed consent for the use of pancreatic tissue in scientific research was obtained from all patients. All details about collection and preparation of human fat pads and in vitro adipocytes differentiation are given in Supplementary information and Suppl. Table 1 and 2.

## Results

### Diet effects on metabolic traits and characterisation of pWAT, eWAT and iWAT of C57BL/6 mice

**Figure 1:**
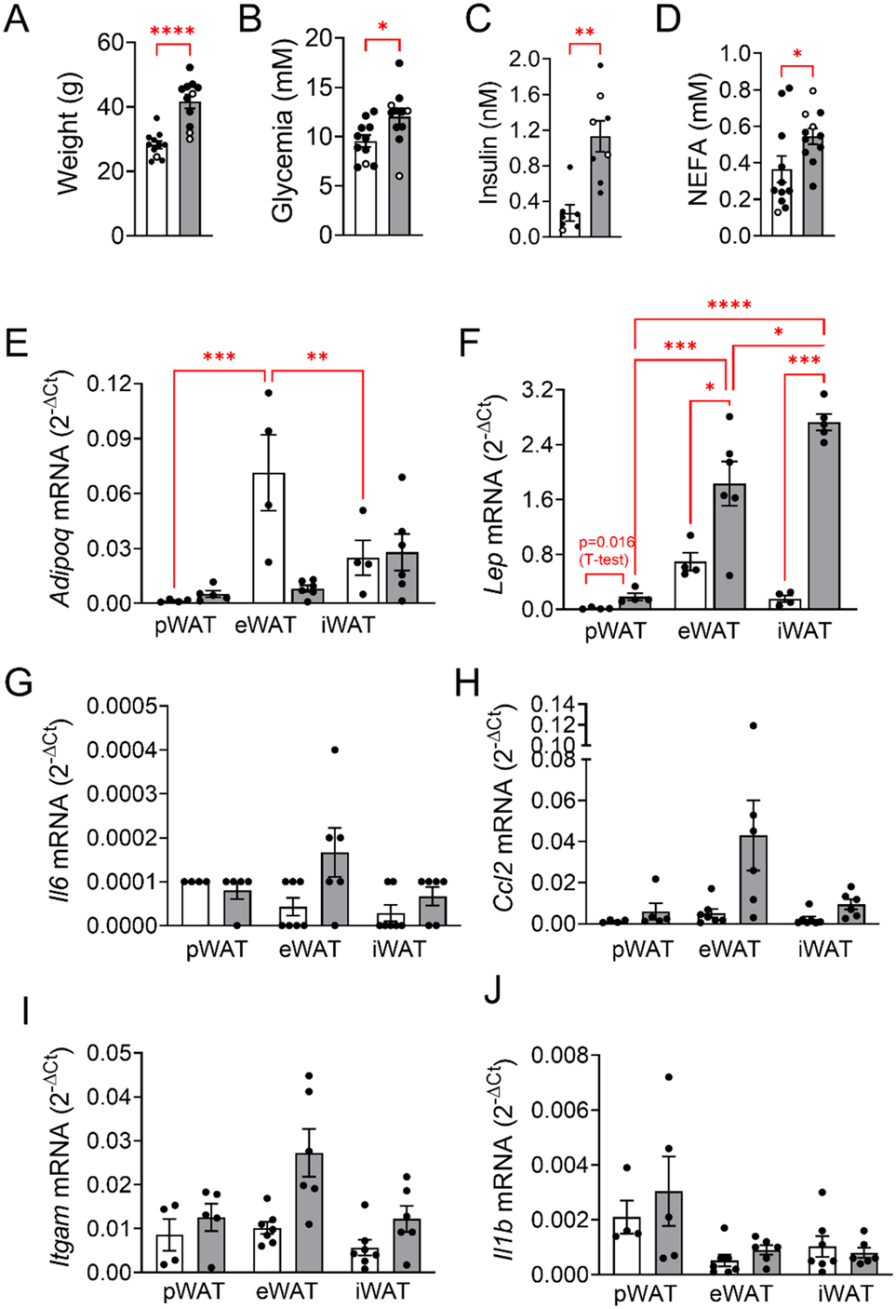
Effects of diet on metabolic traits and adipose tissue characteristics. Mice were fed CD (white bars) and HFD (grey bars). Blood and tissue were collected from male (black dots) and female (white dots) mice at age 24 ± 1.5 weeks (*n* = 7–11/group) and analysed as described under Material and methods. (**A**) animal weight, (**B**) blood glucose, (**C**) serum insulin, (**D**) serum fatty acids and (**E-J**) relative mRNA levels of (**E–F**) adipokines, (**G**-**H**) chemokines and (**I-J**) inflammation markers of pancreatic (pWAT), epididymal (eWAT) and inguinal (iWAT) adipose tissue expressed as 2^−ΔCt^ relative to *Rps13* mRNA (housekeeping gene) are presented as means ± SEM of n individual observations as indicated by the symbols in each plot. *p < 0.05, **p < 0.01, ***p < 0.001, ****p < 0.0001 denote significant differences.

Mice were fed chow (CD) or high-fat diet (HFD) in order to examine obesity-induced alterations of cytokine production and lipolytic activity of pancreatic fat (pWAT) and, in comparison, of visceral, epididymal (eWAT) and subcutaneous, inguinal (iWAT) fat. In comparison to CD, HFD fed mice gained more weight (41.6 ± 2.1 g vs 28.2 ± 1.2 g, *n* = 11 each group, *p* < 0.001) and displayed higher blood glucose (12.1 ± 0.8 mM vs 9.5 ± 0.6 mM, *n* = 11 each group, *p* < 0.05), serum insulin (1.13 ± 0.17 nM, n = 8 vs 0.27 ± 0.09 nM, n = 7, p < 0.05) and NEFA levels (0.55 ± 0.04 mM vs 0.37 ± 0.07 mM, *n* = 11 each group, *p* < 0.05) in the fed state (Fig. 1A-D). Blood triglyceride concentration was not changed (HFD: 1.15 ± 0.08 mM vs CD:1.18 ± 0.12 mM, *n* = 11, data not shown). The pancreatic fat pads (pWAT) of both CD and HFD fed animals are considerably smaller than the other fat pads, iWAT and eWAT Suppl. Fig. 1A). Of note, the adipocytes of HFD pWAT were larger that those of CD pWAT pads (Suppl. Fig. 1B-E).

In CD fed mice, the relative mRNA levels (2^−ΔCt^) of *Adipoq (*adiponectin; 0.002 ± 0.0004, *n* = 4) and *Lep* (leptin; 0.015 ± 0.0068, *n* = 4) were at least 10-times lower than the respective mRNA levels of eWAT (*Adipoq*: 0.071 ± 0.021, *n* = 4 and *Lep:* 0.696 ± 0.129, *n* = 4) and iWAT (*Adipoq*: 0.025 ± 0.01, *n* = 4 and *Lep*: 0.157 ± 0.05, *n* = 4;Fig. 1E,F). HFD increased the mRNA levels of *Lep* in all three fat depots, but pWAT *Lep* mRNA level was still 10-times lower than those of eWAT and iWAT. In parallel, *Adipoq* mRNA levels were slighlty increased in HFD pWAT, while decreased up to eightfold in HFD eWAT. Thus, HFD increased expression of two consecrated adipocyte markers in pWAT.

Since obesity correlates with low grade inflammation and obese adipose tissue can be heavily infiltrated by immune cells, the mRNA levels of specific immune cell markers (*Itgam* and *Il1b*) and of tissue chemokines (*Il6* and *Ccl2*) were quantified in pWAT, eWAT and iWAT (Fig. 1G-J). None of these inflammation markers were significantly altered by HFD compared to CD. Of note, the mRNA levels of *Il6* and *Il1b* were extremely low. These results suggest that macrophage invasion and cytokine production were not stimulated by HFD.

**Figure 2:**
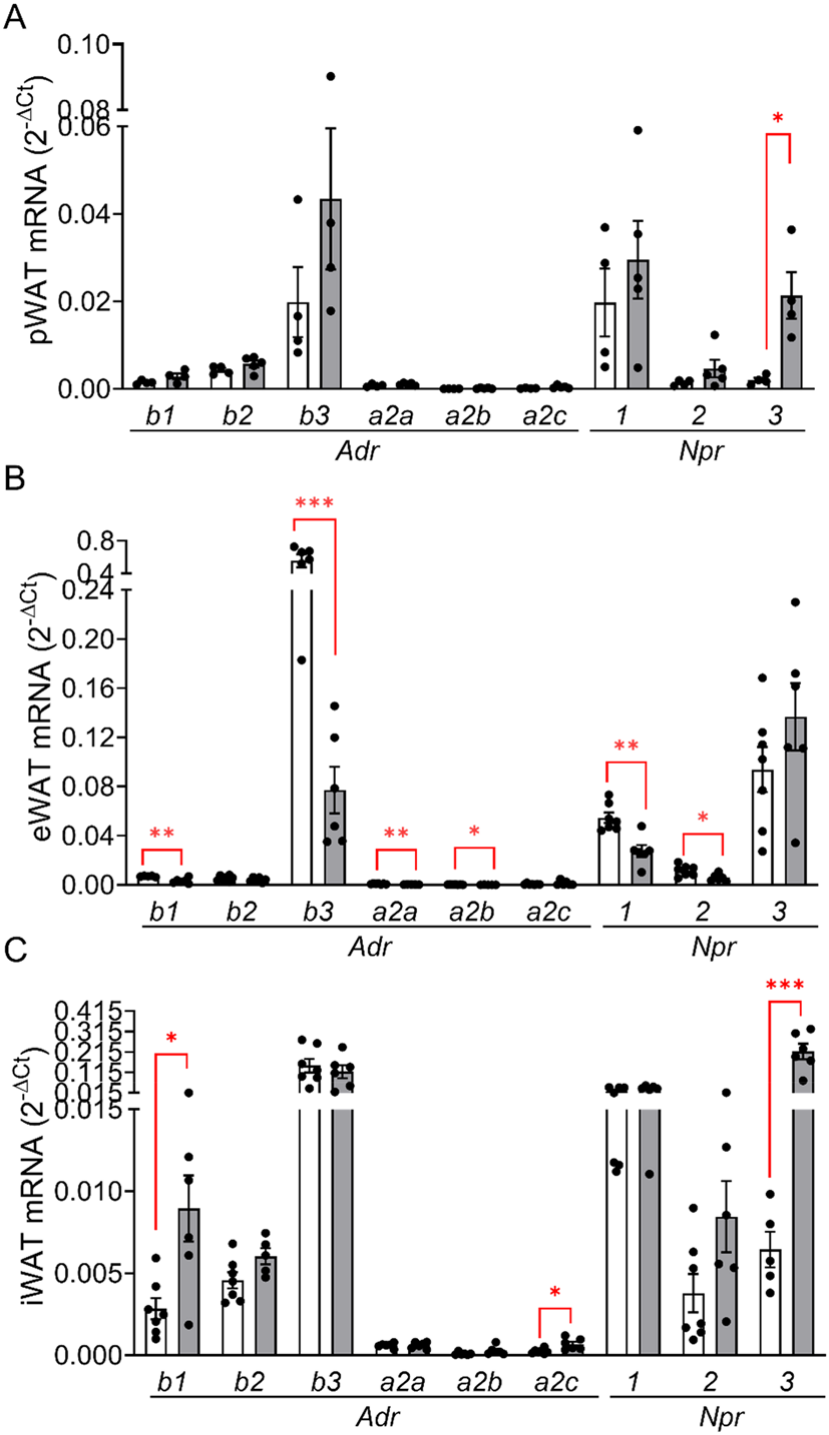
Expression of adrenergic and natriuretic peptide receptors in pWAT, eWAT and iWAT. Relative receptor expression (relative to *Rps13*) in (**A**) pWAT, (**B**) eWAT and (**C**) iWAT was quantified in the fat pads used in Fig. 1E-J. Results are presented as means ± SEM of *n* = 4–7 individual observations. *p < 0.05, **p < 0.01, ***p < 0.001 denotes a significant diet effect.

Next, we examined the expression of receptors known to regulate lipolysis and NEFA production. Beta-adrenoceptor mRNA levels (especially *Adrb3*) were highly abundant, *Npr1-3* (natriuretic peptide receptors) mRNA levels were detectable, whereas all alpha2-adrenoceptor mRNA levels (*Adra2a*, *Adra2b* and *Adra2c*) were very low in all three fat pads (Fig. 2A-C).

Of note, in human pancreatic fat pads beta-adrenoceptors (especially *Adrb2*) were also highly expressed, in contrast to the in vitro differentiated human pancreatic adipocytes that do not express beta-adrenoceptors but NPRs (Suppl. Fig.2 and Ref.^14^). This expression pattern suggests that, next to ANP, sympathetic stimulation may increase lipolysis also in human pancreatic fat.

### Diet effect on lipolysis of pWAT, eWAT and iWAT

**Figure 3:**
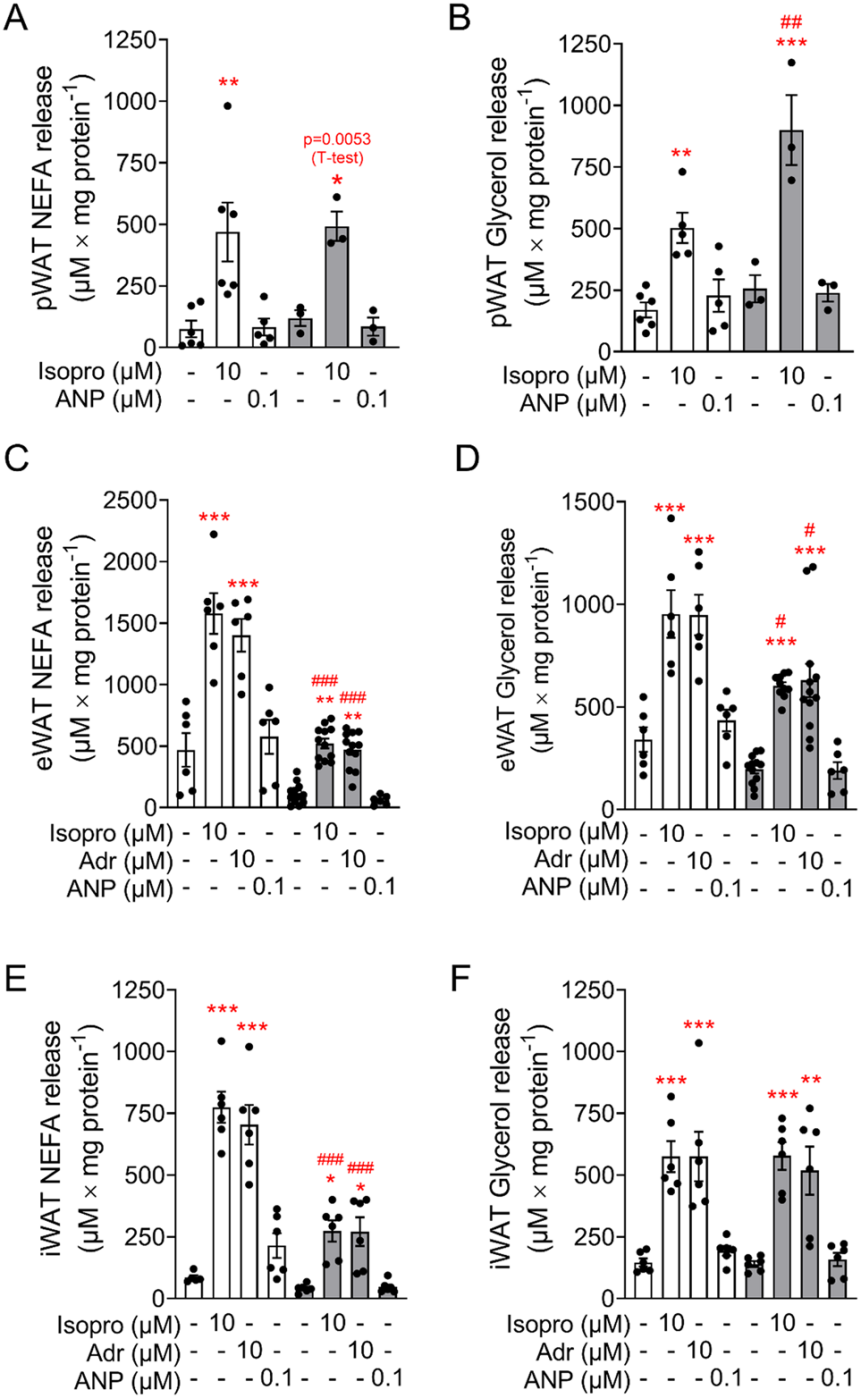
Isoproterenol stimulates lipolysis in adipose tissue. (**A**, **B**) pWAT, (**C**, **D**) eWAT and (**E**, **F**) iWAT pads from CD (white bars) and HFD (grey bars) mice were isolated and incubated as described under Material and methods. (**A**, **C**, **E**) Fatty acids release and (**B**, **D**, **F**) glycerol release in response to isoproterenol (Isopro), atrial natriuretic peptide (ANP) and adrenaline (Adr) are expressed as means ± SEM of *n* = 3–6 independent observation.**p < 0.01, ***p < 0.001 denotes significant stimulation vs the respective control; #*p* < 0.05, ##*p* < 0.01, ###*p* < 0.001 denotes a significant diet effect at the same condition.

The lipolytic capacity of CD (white bars) and HFD (grey bars) pWAT, eWAT and iWAT pads was estimated by determination of NEFA and glycerol accumulation (normalized to tissue amount) in the incubation buffers. Lipolysis was stimulated with isoproterenol, a selective beta-adrenergic agonist, adrenaline and ANP (Fig. 3A-F). Under basal, unstimulated condition NEFA secretion of pWAT was about 16% of that of eWAT (75 ± 37 µM/mg protein (*n* = 5) and 469 ± 136 µM/mg protein (*n* = 6), respectively), a discrepancy which disappeared in HFD fed animals (119 ± 32 µM/mg protein (*n* = 3) and 113 ± 25 µM/mg protein (*n* = 12)) (Fig. 3A,C). Isoproterenol (10 µM) stimulated lipolysis 4- to sixfold in pWAT, eWAT and iWAT (Fig. 3). Adrenaline (10 µM) was as effective as isoproterenol in stimulating lipolysis of eWAT and iWAT (Fig. 3C,E). While NEFA release of eWAT and iWAT was significantly lower upon HFD, NEFA release of pWAT was not altered by HFD (Fig. 3A,C,E). Isoproterenol and adrenaline augmented also glycerol release, confirming that the increase of NEFA concentration in the incubation solution results from triglyceride lipolysis (Fig. 3B,D,F). Of note, isoproterenol-stimulated glycerol release of HFD pWAT was significantly higher compared to that of CD pWAT (Fig. 3B). The glycerol release of HFD eWAT mirrored the NEFA profile, and was lower than in CD eWAT. Glycerol release of iWAT was unaltered by diet (Fig. 3C-F). These results suggest that NEFA release from pWAT, in contrast to eWAT, is not affected by HFD, and that beta-adrenergic stimulation raises the NEFA load in the pancreatic environment.

### Effect of diet and adrenergic stimulation on adipose tissue-islet crosstalk

**Figure 4:**
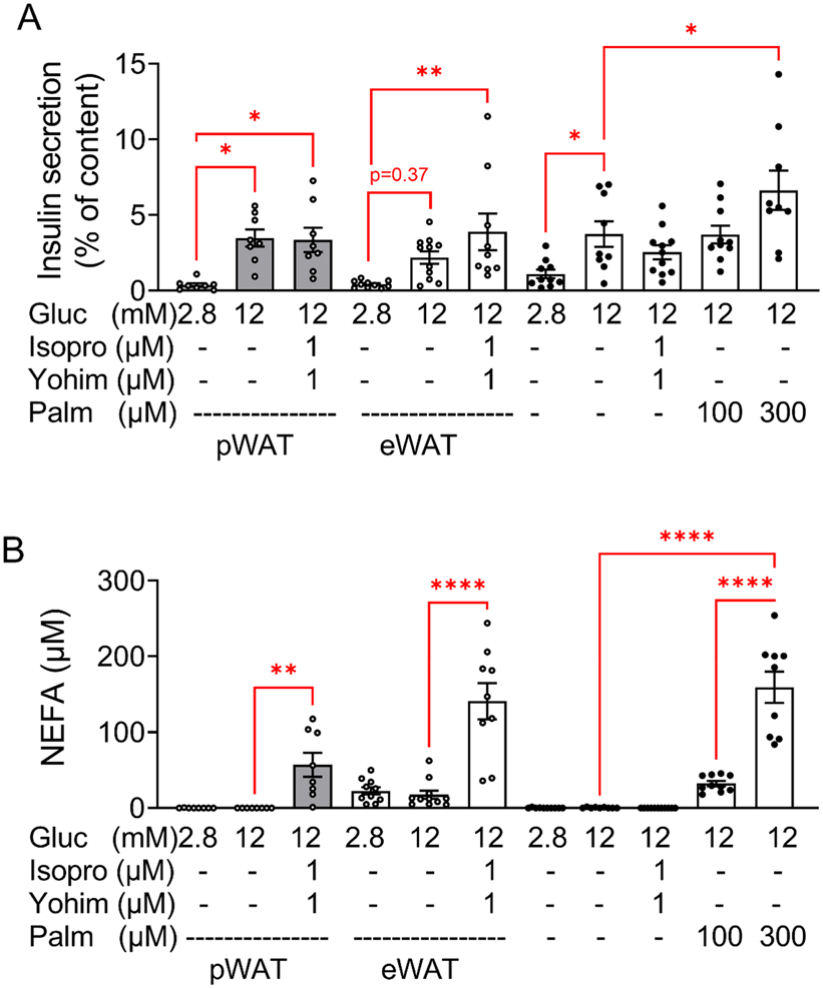
Short term co-incubation of pWAT and eWAT of CD mice with isolated islets of CD mice. Adipose tissue was pre-incubated and islets were isolated, cultured overnight and co-incubated as described under Material and methods. Glucose (Gluc), isoproterenol (Isopro), yohimbine (Yohim), palmitate (Palm) were added as indicated. (**A**) Insulin and (**B**) NEFA were measured in the supernatant. Results are expressed as means ± SEM of *n* = 8–12 observations of 3 independent experiments.

To identify a putative fat-islet crosstalk in vitro, we established a co-incubation system using islets isolated from CD fed mice and pWAT/eWAT from CD and HFD mice. Lipolysis was initiated before the addition of islets to the adipose tissue samples for 1 h co-incubation. Lipolysis was stimulated with 1 µM isoproterenol, a concentration as effective as 10 µM (Suppl. Fig. 3A). Furthermore, 1 µM yohimbine (alpha2-adrenoceptor antagonist) was used to avoid activation of inhibitory alpha2-adrenoceptors on beta cells. Triacsin C, an inhibitor of fatty acids re-uptake was omitted, as it interfered with GSIS, and its omission had no effect on NEFA accumulation in the medium (Suppl. Fig. 3B,C). In control islets (no co-incubation), glucose (12 mM) stimulated insulin secretion 3- and twofold, in the absence and presence of isoproterenol + yohimbine, respectively (Fig. 4A). Palmitate (300 µM) further augmented GSIS 1.8-fold (Fig. 4A). In the presence of pWAT, basal insulin release (at 2.8 mM glucose) was reduced resulting in a ninefold stimulation of secretion by glucose. Stimulation of pWAT lipolysis with isoproterenol + yohimbine, and NEFA accumulation (57 ± 15 µM) in the incubation buffer did not affect GSIS.

In the presence of eWAT, GSIS reached significance only in the presence of isoproterenol + yohimbine. Under this condition, the level of NEFAs in the co-incubation buffer increased to a concentration (140 ± 23 µM) similar to that created by exogenously added palmitate (159 ± 20 µM) that augmented GSIS (Fig. 4B, columns 6 and 11).

These results show that short term co-incubation of isolated islets with CD pWAT had no impact on GSIS. However, these results also suggest that lipolysis-originating NEFA improve GSIS when their concentration raises sufficiently.

**Figure 5:**
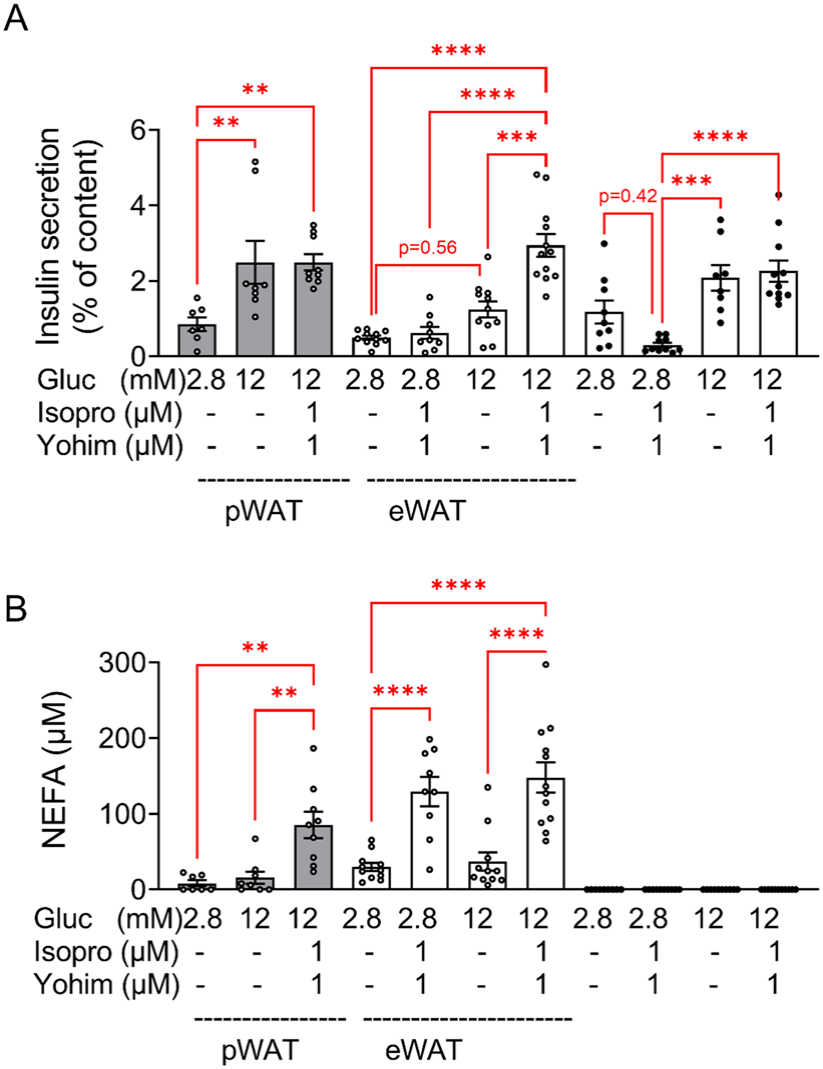
Short term co-incubation of pWAT and eWAT of HFD mice with isolated islets of CD mice. Adipose tissue was preincubated and islets were isolated, cultured overnight and co-incubated as described under Material and methods. Glucose (Gluc), isoproterenol (Isopro), yohimbine (Yohim) were added as indicated. (**A**) Insulin and (**B**) NEFA were measured in the supernatant. Results are expressed as means ± SEM of *n* = 8–12 observations of 3 independent experiments.

Since obesity alters the physiology of adipose tissue, additional experiments were performed with pWAT and eWAT isolated from HFD mice in order to assess whether HFD feeding alters the islet-fat crosstalk. In control islets (no co-incubation) glucose (12 mM) increased insulin secretion 1.8-fold without reaching significance due to the variable basal secretion at 2.8 mM glucose (Fig. 4A, column 8 and 10). In the presence of isoproterenol + yohimbine GSIS turned significant (ninefold) due to a reduced basal secretion (Fig. 5A, column 9–11).

When islets were co-incubated with HFD pWAT, glucose (12 mM) stimulated insulin secretion threefold. Similar to the co-incubation with CD pWAT, isoproterenol + yohimbine did not augment GSIS in spite of increasing NEFA release (85 ± 17 µM) (Fig. 5A,B).

In the presence of HFD eWAT, insulin secretion was 2.4-fold higher at 12 mM compared to 2.8 mM glucose, and 4.8-fold in the presence of isoproterenol + yohimbine. Beta-adrenergic stimulation of eWAT increased NEFA concentration at 2.8 and 12 mM glucose to 129 ± 19 µM and 148 ± 19 µM, respectively. As expected, high NEFA did not stimulate basal insulin secretion but augmented GSIS (Fig. 5B, columns 4–7).

These results suggest that the beneficial effect of HFD eWAT on GSIS (12 mM glucose) is mediated by the released NEFA via lipolysis (Fig. 5, columns 6 and 7), since isoproterenol + yohimbine had no effect on GSIS (at 12 mM glucose) in the absence of eWAT (Fig. 4A columns 8 and 9; Fig. 5A, columns 10 and 11).

Thus, increased sympathetic activity stimulates adipose tissue lipolysis and thereby may augment the NEFA load of tissue microenvironement and favour GSIS.

**Figure 6:**
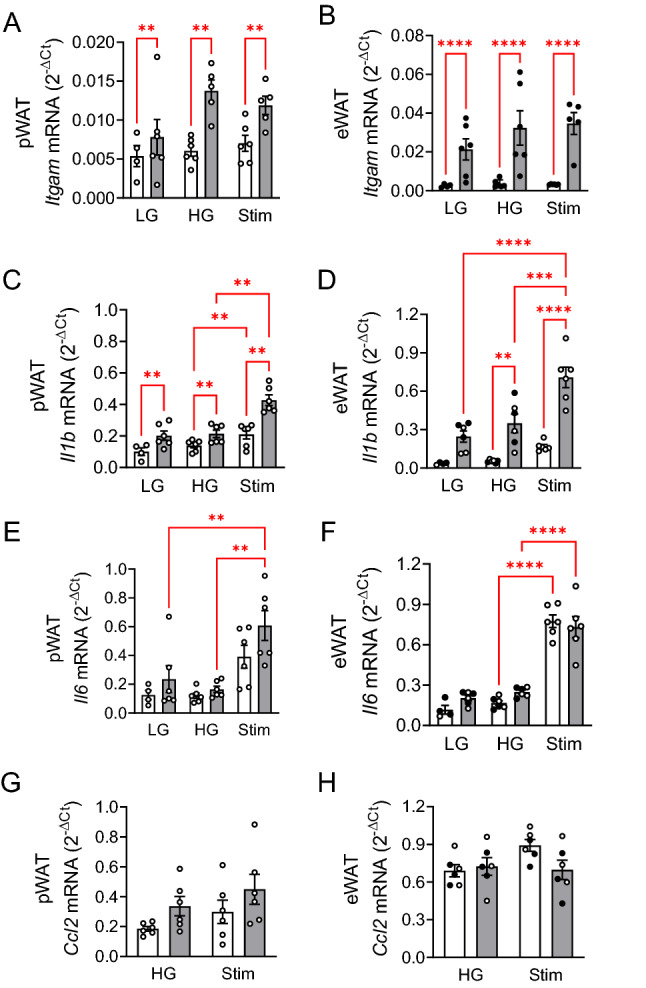
Beta-adrenergic stimulation increases *Il1b* and *Il6* mRNA levels in adipose tissue. Cellular mRNA was isolated from (**A, C, E, G**) pWAT and (**B, D, F H**) eWAT pads used in the co-incubation experiments shown in Fig. 4 (CD mice, white bars) and Fig. 5 (HFD mice, grey bars) under low (2.8 mM, LG) and high (12 mM, HG) glucose and stimulation (12 mM glucose + Isopro + Yohim, Stim). Relative mRNA levels to *Rps13* are presented as means ± SEM of the number of individual observations shown in each bar. *p < 0.5, **p < 0.05, ***p < 0.05, ****p < 0.001 denote significant differences.

In order to assess the effect of beta-adrenergic stimulation on the inflammatory status of eWAT and pWAT, we measured the level of inflammatory markers in the fat pads following their co-incubation with islets (Fig. 6). In contrast to the freshly isolated fat tissue samples (Fig. 1), HFD significantly increased the mRNA level of *Itgam* and *Il1b* both in pWAT and eWAT, irrespective of the incubation condition (2.8 mM (LG) vs 12 mM glucose (HG) vs 12 mM glucose + isoproterenol + yohimbine (Stim); Fig. 6A-D). HFD had no impact on the mRNA levels of *Il6* and *Ccl2* (Fig. 6, compare white and grey columns of the respective condition, LG, HG and Stim). Noteworthy, isoproterenol (+ yohimbine) significantly increased *Il1b* and *Il6* mRNA levels of pWAT and eWAT (Fig. 6, compare HG and Stim of respective diet, white or grey columns). Thus, beta-adrenergic stimulation increases cytokine (IL-1beta and IL-6) production in adipose tissue (pWAT and eWAT).

## Discussion

Our data demonstrate that activation of adrenergic receptors, part of sympathetic nervous system (SNS), augments IL-1beta production and NEFA release in pancreatic fat. These events define the autonomic nervous system as a modulator of the islet-adipose tissue crosstalk. Previous observations suggest that obesity, in particular visceral fat accumulation, leads to increased activity of SNS in humans^26–28^.

The in vitro assessment of fat-islet crosstalk was challenging owing to several reasons. Firstly, in vitro differentiated pancreatic adipocytes lost their adrenoceptors expression during differentiation and were unresponsive to adrenaline. To circumvent such cell culture-originating shortcomings we used freshly isolated fat pads from CD and HFD mice, that allow also examination of effects induced by the tissue-resident immune cells. Secondly, the optimal incubation conditions for in vitro lipolysis and insulin secretion differ considerably. Thus, fatty acids are only sparingly soluble in aqueous solution and require high concentrations of albumin to dissolve. Therefore, the lipolysis buffer contained a high concentration (2%) of fatty acid-free BSA in order to dissolve the fatty acids released during lipolysis. Such a condition is not optimal for islet function, as fatty acid-free BSA impairs GSIS^29,30^. In addition, long chain fatty acids augment GSIS mainly via activation of FFA1, a receptor activated only by free, unbound fatty acids^31–33^. Therefore, after 1 h of lipolysis and before addition of islets to the system, albumin concentration was reduced to 0.5% in order to enable the lipolysis-originating NEFA to activate islets’ FFA1. We considered NEFA-mediated stimulation of GSIS as readout of fat-islet crosstalk. Thirdly, adrenaline stimulates lipolysis through beta-adrenoceptors, whereas it inhibits GSIS via alpha2-adrenoceptors^34,35^. For this reason, we stimulated lipolysis of the co-cultured fat pads only with the beta-adrenoceptor agonist isoproterenol. Such incongruous requirements render a long-term islet-fat co-culture rather difficult and raise the question about the necessity of spatial separation of the two tissue compartments in organ-on-a-chip-devices.

According to our results, lipolysis of CD pWAT was 3– to fourfold lower than in CD eWAT, mirroring the lower levels of Adipoq and Lep mRNA in pWAT, as previously described^8^.

On the other side, the intrapancreatic insulin concentration is high even under non-insulin-resistant conditions, and the low lipolytic rate of CD pWAT might reflect its metabolic memory. We previously found that human T2D pancreatic adipocytes have impaired lipolysis and higher Lep/Adipoq ratio^14^. However, HFD neither altered lipolysis nor impaired Adrb3, Adipoq and Lep expression of pWAT, and thus HFD pWAT did not reflect the phenotype of human T2D adipocytes. Interestingly, a previous work suggested that mouse pancreatic fat might be protected from some deleterious effects of HFD^8^. Since the amount of locally secreted NEFAs depends also on the number of infiltrating adipocytes, a pronounced pancreatic steatosis would considerably augment the NEFA load, in spite of the rather low lipolytic capacity of pWAT. On the contrary, HFD reduced both basal and stimulated lipolytic rate of eWAT. This reduction mirrors downregulation of Adrb3 and Adipoq, and upregulation of Lep, since leptin increases and adiponectin decreases lipolysis^36^.

Isoproterenol and adrenaline stimulated lipolysis with equal efficiency, so that alpha2-adrenoceptor inhibition of lipolysis, as previously described in isolated human subcutaneous adipocytes, can be ruled out^37,38^. Our results are in line with the high expression of stimulatory beta-adrenoceptors and a much lower expression of inhibitory alpha2-adrenoceptors in mouse fat pads. ANP did not stimulate lipolysis, in agreement with previous observation that ANP stimulates lipolysis in human but not mouse adipocytes due to the higher expression of the ANP-degrading NPR3 receptor in mice^39^. The pattern of NEFA/glycerol release in mouse pWAT, eWAT and iWAT is considerably different from that of in vitro differentiated primary human adipocytes, where ANP but not adrenaline stimulated lipolysis^14^. This is in accordance with the lower expression of adrenoceptors in in vitro differentiated adipocytes than in human fat pads (Suppl. Fig. 1). Thus, the stress factors adrenaline and ANP, that increases with intravasal volume overload and congestive heart failure, may synergistically activate lipolysis of human pWAT^40,41^.

Using islets from an obese, diabetes prone mouse model, a previous work proposed that insulin hypersecretion is linked to fatty acid released from pancreatic adipocytes^13^. The authors performed a 48 h co-culture of isolated islets with in vitro differentiated adipocytes. Such a long-term exposure of isolated islets to fatty acids, and high glucose, evokes a persistent hypersecretion, resulting in reduced insulin content and defective glucose responsiveness^42^. We used here a 1 h co-incubation of isolated islets with fat pads to examine the short-term effect of adipose tissue on GSIS. CD/HFD pWAT had no effect on GSIS, while eWAT impaired insulin secretion in the absence of stimulated NEFA release. Considering that the fat pads are composed of different cell types, other factors, in addition to NEFA, may impact on insulin secretion. Stimulation of lipolysis of HFD eWAT augmented GSIS, in spite of upregulated *Il1b mRNA,* suggesting that the rapidly acting positive NEFA effect dominated over the negative, cytokine-mediated one. The missing effect of pWAT on GSIS is in accordance with previous findings showing that overnight incubation of murine islets with pWAT preserves GSIS^8^. However, we cannot rule out that the FFA concentration in the co-incubation buffer remained under the level necessary to stimulate FFA1 and GSIS. These experiments were limited by the very low amount of pWAT even after HFD feeding (~ 70 mg/mouse). Nevertheless, we can conclude that an increase of NEFA in the immediate proximity of islets augments GSIS.

Since fat tissue contains a considerable number of immune cells, we assessed the mRNA levels of markers of macrophage infiltration, i.e. Itgam, Il1b, Il6 and Ccl2 in freshly isolated fat pads. While *Itgam* and *Ccl2* mRNAs were highly variable, *Il1b* and *Il6* mRNA levels were extremely low suggesting that their production was not induced in vivo, contrary to a previous work which detected increased immune cell infiltration of mouse pWAT compared to eWAT/iWAT^8^. However, after in vitro incubation of fat pads, *Itgam and Il1b* mRNA were upregulated in HFD pWAT/eWAT, indicating a diet-dependent and treatment-induced activation of the resident immune cells. Such a beta-adrenergic stimulation of IL-1beta and IL-6 production has been described in a murine macrophage cell line^43^. Furthermore, long term exposure of beta-cells to IL-1beta and NEFAs promotes loss of beta-cell mass and function^21,44–48^. Of note, only saturated long chain fatty acids induce beta-cell death, preferentially under hyperglycaemic conditions, while lipolysis generates both saturated and unsaturated NEFAs^10,46^.

In summary, our data suggest that sympathetic activation of pWAT may increase tissue concentration of NEFA and cytokines. A dysregulated SNS activity during obesity has been reported and might contribute to hyperglycaemia, as insulin secretion is directly inhibited via beta-cell alpha2-adrenergic receptors^26,35,49,50^. In such a situation NEFAs would stimulate glucagon release which further mobilises hepatic glucose production thereby increasing insulin demand and promoting beta-cell exhaustion^34,51,52^.

## Conclusion

Our data suggest that a sympathetic activity–induced augmentation of NEFA and cytokine load within the islet proximity may contribute to obesity-linked transition from insulin hypersecretion to beta-cell failure.

## Supplementary Information

Supplementary Information.

## Data Availability

The datasets used and/or analysed in the current study are available from the corresponding author on reasonable request.

## Abbreviations

ANP: Atrial natriuretic peptide
Adipoq: Adiponectin
BSA: Bovine serum albumin
CD: Standard chow diet
ELISA: Enzyme-linked immunosorbent assay
eWAT: Epididymal white adipose tissue
FCS: Foetal calf serum
GSIS: Glucose-stimulated insulin secretion
HFD: High-fat diet
IFG: Impaired fasting glucose
IGT: Impaired glucose tolerance
iWAT: Inguinal white adipose tissue
KRB: Krebs–Ringer bicarbonate buffered saline
Lep: Leptin
NEFAs: Non-esterified fatty acids
NGT: Normal glucose tolerance
pWAT: Pancreatic white adipose tissue
SNS: Sympathetic nervous system
TLR4: Toll-like receptor 4
T2D: Type 2 diabetes mellitus
