# Supplementary material for: Effects of adrenergic-stimulated lipolysis and cytokine production on in vitro mouse adipose tissue–islet interactions
Source: Sci Rep. 2022 Sep 22;12:15831. doi: 10.1038/s41598-022-18262-0 (PMC9499973; doi:10.1038/s41598-022-18262-0)
Supplement: Supplementary file 1 — Supplementary Information. [file 41598_2022_18262_MOESM1_ESM.docx]

**Supplementary Information**

**Human pancreatic resections**

Pancreatic fat pads were obtained from 29 partially pancreatectomized patients (PPP) undergoing pancreatic surgery (20 males, 9 females, age 41-82 years, BMI 19-33 kg/cm²). Subject characteristics are given in Suppl. Table 2. Fasting blood samples were collected prior to surgery to analyze the metabolic status of the patient and to measure insulin secretion and sensitivity. Metabolic status classification was performed following the American Diabetes Association (ADA) guidelines^1^. The patients were defined as normal glucose tolerant (NGT) with fasting glucose <5.6 mM and HbA1c <5.7%, prediabetic (PD) with fasting glucose between 5.6 mM-6.9 mM or HbA1c between 5.7%-6.4%, and diabetic (T2D) with fasting glucose ≥7.0 mM or HbA1c ≥6.5%. Insulin secretion and sensitivity were measured using the HOMA2 method^2^. Insulin secretion (HOMA2%B) was computed from fasting glucose and C-peptide levels. Insulin sensitivity (HOMA2%S) was assessed using fasting glucose and specific insulin levels. Beta-cell function adjusted for insulin sensitivity, HOMA2 disposition index, was calculated with the following formula: (HOMA2%B x HOMA2%S)/10,000.

**Human pancreatic preadipocyte isolation and *in vitro* differentiation**

Fat biopsies were immediately processed for RNA extraction or used for preadipocyte isolation as previously described^3^. Isolated preadipocytes were expanded in alpha-MEM/Ham’s mixture F12 (1:1) (Lonza) supplemented with 2 mM L-glutamine, 20% (v/v) FCS, 1% (v/v) chicken embryo extract (Sera Laboratories International, Horsted Keynes, UK), 1% (v/v) penicillin/streptomycin, and 0.5 mg/ml amphotericin B. Adipocyte differentiation was achieved after 14 days cultivation in DMEM/Ham’s mixture F12 (1:1) with 2 mM L-glutamine, 17 mM pantothenate, 1 mM biotin, 10 mM troglitazone, 0.001 mM insulin, 2 mg/ml apotransferin, 0.5 mg/ml amphotericin, 5% (v/v) FCS and 1% (v/v) penicillin/streptomycin. During the first 7 days of differentiation, medium was supplemented with 0.5 mM IBMX (3-isobutyl-1-methyl-xanthine), 0.001 mM cortisol and 0.05 mM indomethacin. During the second week of differentiation, adipocytes were cultured in DMEM/Ham’s mixture F12 without supplements.

**Suppl. Table 1.** Mouse and human primers and probes for PCR amplification.

| **Gene** | **Forward primer sequence** | **Reverse primer sequence** | **Probe** | | |
| --- | --- | --- | --- | --- | --- |
| **Mouse** | | | | |  |
| *Rps13* | 5‑TGCTCCCACCTAATTGGAAA-3 | 5-CTTGTGCACACAACAGCATTT-3 | #110 | | |
| *Adrb1* | 5-ACCCGAGTGGAAACTAGGC-3 | 5‑ACCGGAAAGCCAGGTGATA-3 | #58 | | |
| *Adrb2* | 5‑GCATGGAAGGCTTTGTGAAC-3 | 5-CTTGGGAGTCAACGCTAAGG-3 | #98 | | |
| *Adrb3* | 5-CAGCCAGCCCTGTTGAAG-3 | 5‑CCTTCATAGCCATCAAACCTG-3 | #13 | | |
| *Adra2a* | 5‑TAGAACTGACTTTTCTTCCGTTCTC-3 | 5‑AACATACACGCTCTTCTTCAAGC-3 | #82 | | |
| *Adra2b* | 5‑AGCACCTGTGGTTCTCCTTG-3 | 5-CAGCAACCAGCACTAGACCA-3 | #83 | | |
| *Adra2c* | 5-CTTCAGGCAATGACCCTCTG-3 | 5‑AGAGCTGTCCAGGACGTCAG-3 | #85 | | |
| *Npr1* | 5‑TGGAGACACAGTCAACACAGC-3 | 5-CGAAGACAAGTGGATCCTGAG-3 | #60 | | |
| *Npr2* | 5-TGAGCAAGCCACCCACTT-3 | 5‑AGGGGGCCGCAGATATAC-3 | #21 | | |
| *Npr3* | 5‑CTCTTCAACAGTTCTTCCTACGG-3 | 5‑GCTTGTTTAGCTTCAGAGTCGTG‑3 | #55 | | |
| *Ins2* | 5‑GAAGTGGAGGACCCACAAGT-3 | 5-AGTGCCAAGGTCTGAAGGTC-3 | #32 | | |
| *Gcg* | 5-CCAGTGATGTGAGTTCTTACTTGG-3 | 5‑CAATGGCGACTTCTTCTGG-3 | #27 | | |
| *Sst* | 5‑CCCAGACTCCGTCAGTTTCT-3 | 5-GGGCATCATTCTCTGTCTGG-3 | #53 | | |
| *Adipoq* | 5-GAGGGAGAAAAGGCGAGCTT-3 | 5-GGGTTCCTCTGCTTGTTCCA-3 | #10 | | |
| *Lep* | 5-ACATACCGCATTTCAGGGCA-3 | 5-CCCAGGTATCCCGTGTCAAC-3 | #86 | | |
| *Il6* | 5-GCTACCAAACTGGATATAATCAGGA-3 | 5-CCAGGTAGCTATGGTACTCCAGAA-3 | #6 | | |
| *Il1b* | 5-AGTTGACGGACCCCAAAAG-3 | 5-AGCTGGATGCTCTCATCAGG-3 | #38 | | |
| *Il8* | 5-TGCTCAAGGCTGGTCCAT-3 | 5-GACATCGTAGCTCTTGAGTGTCA-3 | #18 | | |
| *Ccl2* | 5-CATCCACGTGTTGGCTCA-3 | 5-GATCATCTTGCTGGTGAATGAGT-3 | #62 | | |
| **Human** | | | |  |  |
| *RPS13* | 5-CCCCACTTGGTTGAAGTTGA-3 | 5-ACACCATGTGAATCTCTCAGGA-3 | #68 | | |
| *ADRB1* | 5-GTGGAAGATGGGTGGGTTAG-3 | 5-GAGCCACGATGATCGATTTTA-3 | #7 | | |
| *ADRB2* | 5-CCATGTCCAGAACCTTAGCC-3 | 5-GATCTGCGGAGTCCATGC-3 | #15 | | |
| *ADRB3* | 5-TCTACTCTGCGCTGGCTTTT-3 | 5-GGAAGAAAAGAGTGAGGATTAGGC-3 | #34 | | |
| *ADRA2A* | 5-ACATCCCCAGTTGTTGGTTT-3 | 5-GGGTGGCCCACTAGGAAG-3 | #1 | | |
| *ADRA2B* | 5-GGCTTCCCTTTCTTGAGGAC-3 | 5-AAACGAAAACACCACAAGCA-3 | #72 | | |
| *ADRA2C* | 5-CAGGAGCTTGGCAGAGAGAT-3 | 5-GGAAGGCAAAGGGGTCTC-3 | #1 | | |
| *NPR1* | 5-AGGACGACCTCAGCCACTAC-3 | 5-GGGAGCTGCAGATGTAGATAACTC-3 | #66 | | |
| *NPR2* | 5-TGTGATAAAACTCCACTTTCAACC-3 | 5-TCTCCAGCATCAGCTTTCG-3 | #21 | | |
| *NPR3* | 5-AATTGTCGTGGGGGCTTTA-3 | 5-CGCCTCTCAATGGTTATTCTG-3 | #17 | | |

**Suppl. Table 2.** Characteristics of human pancreatic fat donors.

| **Trait** | **NGT** | **PD** | **T2D** | **p** |
| --- | --- | --- | --- | --- |
| N (% males) | 9 (67) | 11 (82) | 9 (56) | 0.7029 |
| Age (years) | 69.0 (10.0) | 65.0 (10.5) | 66.0 (6.0) | 0.8263 |
| BMI (kg/m²) | 24.5 (4.7) | 23.1 (5.6) | 28.0 (5.7) | 0.6314 |
| Fasting glucose (mmol/l) | 4.9 (0.6) | 5.3 (1.1) | 7.9 (2.1)^***,†††^ | <0.0001 |
| HbA1c (%) | 5.3 (0.6)^3^ | 5.9 (0.3)^‡‡‡^ | 7.9 (1.4)^***,†††^ | <0.0001 |
| Fasting insulin (pmol/l) | 29.0 (27.0) | 48.0 (26.5) | 83.0 (76.0) | 0.0902 |
| Proinsulin (pmol/l) | 2.0 (1.0) | 2.5 (3.3)^4^ | 2.0 (3.0) | 0.3457 |
| C-peptide (µg/l) | 304 (52) | 400 (227) | 356 (208) | 0.3845 |
| HOMA2%B | 77 (3)^1^ | 68 (10)^1^ | 38 (21)^2,*^ | 0.0198 |
| HOMA2%S | 84 (56)^1^ | 113 (56)^1^ | 42 (52)^2^ | 0.2611 |
| HOMA2 disposition index | 0.67 (0.46)^1^ | 0.70 (0.48)^1^ | 0.23 (0.22)^2,‡,§^ | 0.0996 |
| NEFA (µmol/l) | 724 (483) | 662 (393) | 974 (656) | 0.2491 |
| Triglycerides (mg/dl) | 170 (35) | 153 (114) | 172 (50) | 0.8750 |
| Total cholesterol (mg/dl) | 216 (119) | 218 (94) | 204 (41) | 0.1897 |
| HDL cholesterol (mg/dl) | 35 (12) | 39 (16) | 54 (35) | 0.1263 |
| LDL cholesterol (mg/dl) | 161 (65) | 131 (61) | 116 (40)^‡^ | 0.0449 |

Results expressed as median (IQR). ^1^available from 4 subjects; ^2^available from 6 subjects; ^3^available from 8 subjects; ^4^available from 10 subjects. ^*^p<0.05, ^***^p<0.001 vs NGT; ^†††^p<0.001 vs PD; one-way ANOVA followed by Tukey post-testing. ^‡^p<0.05, ^‡‡‡^p<0.001 vs NGT; ^§^p<0.05 vs PD; unpaired t-test. p, one-way ANOVA p-value; NGT, normal glucose tolerance; PD, prediabetes; T2D, type 2 diabetes.


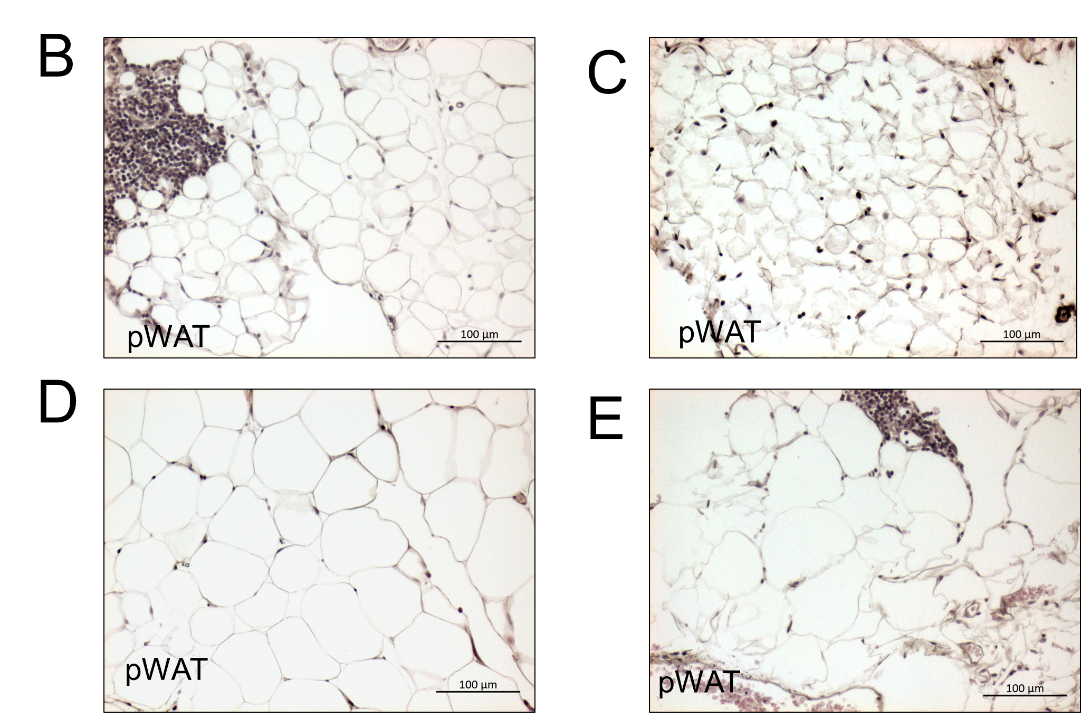

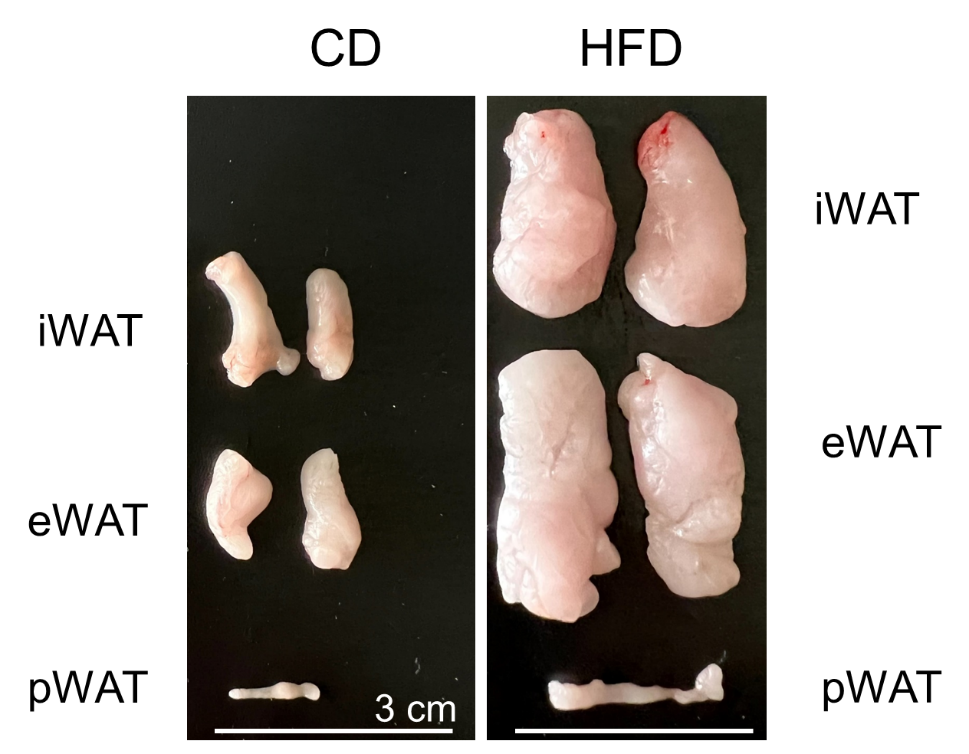


**Suppl. Fig. 1: Mouse pancreas fat is a minor fat depot.** Shown are (A) representative macroscopic images of iWAT, eWAT and pWAT of a CD (left) and HFD (right) male mouse. The weight of the fat pads (n=3 mice/diet) was as follows: iWAT 0.9 ± 0.03 g (CD) and 2.9 ± 0.02 g (HFD); eWAT 1.0 ± 0.07 g (CD) and 2.7 ± 0.1 g (HFD); pWAT 0.005 ± 0.002 g (CD) and and 0.07 ± 0.02 g (HFD), respectively. pWAT was almost absent in CD fed mice. Upon high fat feeding the weight of pWAT increased 14-fold, while for iWAT and eWAT the weight gain was 3-fold and 2.7-fold, respectively. While eWAT and iWAT represent 5-6 % of body weight each, pWAT remained below 0.2 % of body weight in HFD fed mice. (B-E) Representative images (20x) of H&E staining of pWAT from (B,C) two CD male and (D,E) two HFD male mice. Note the increased size of pWAT adipocytes in HFD mice.

**
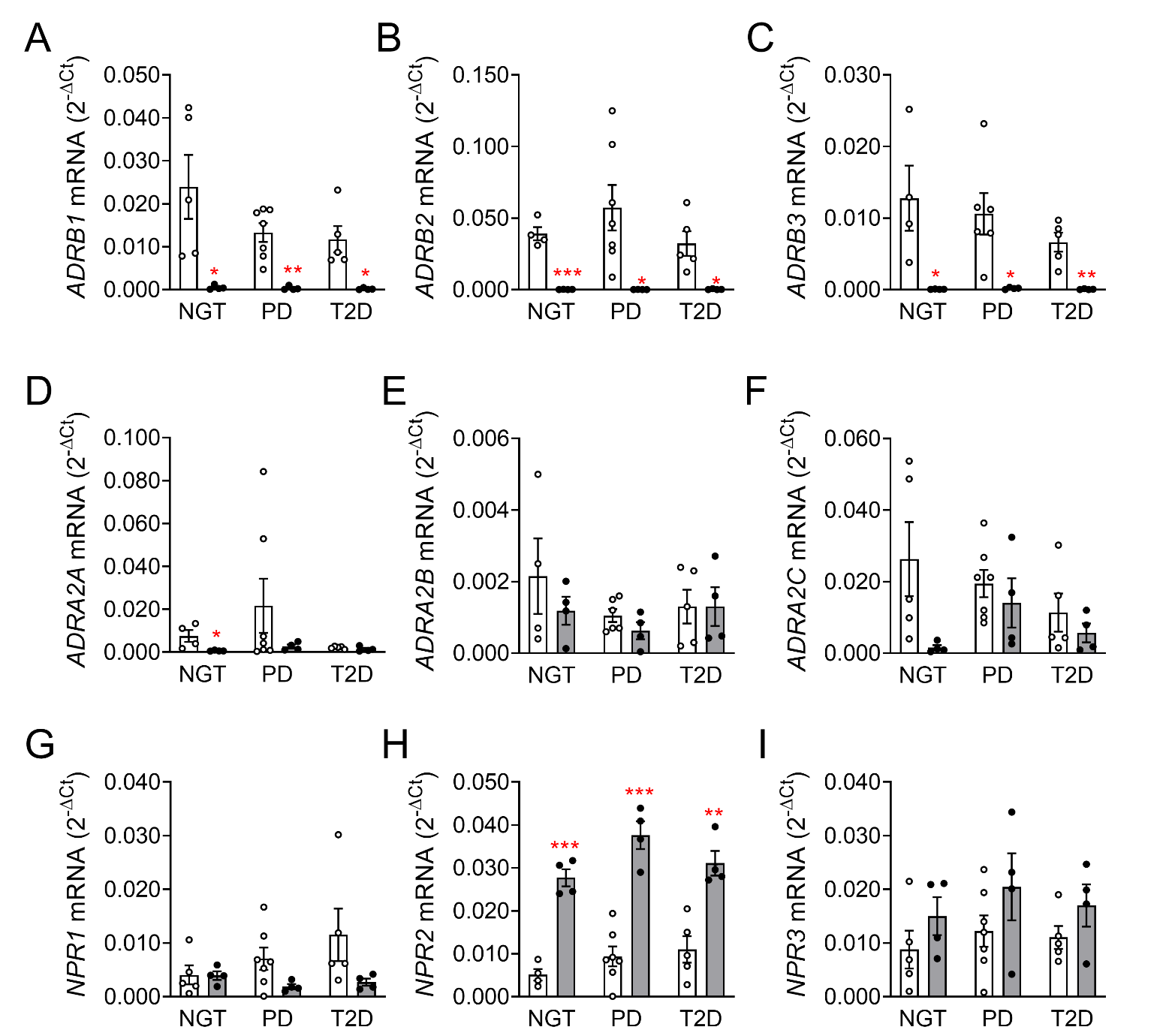
**

**Suppl. Fig. 2: Expression of receptors in human pancreatic fat pads and in vitro differentiated adipocytes.** Fat pads and adipocytes were obtained from patients with NGT, PD, and T2D. mRNA levels of (A-C) beta-adrenergic receptors, (D-F) alpha2-adrenergic receptors, and (G-I) natriuretic peptide receptors in fat pads (○ and white columns) and primary adipocytes (● and grey columns). Relative mRNA levels (to *RPS13*) are expressed as means ± SEM of n=4-7 preparations. *p<0.05, **p<0.01, ***p<0.001 vs fat pads of the respective patient group; t-test. NGT, normal glucose regulation; PD, prediabetic; T2D, type 2 diabetes.


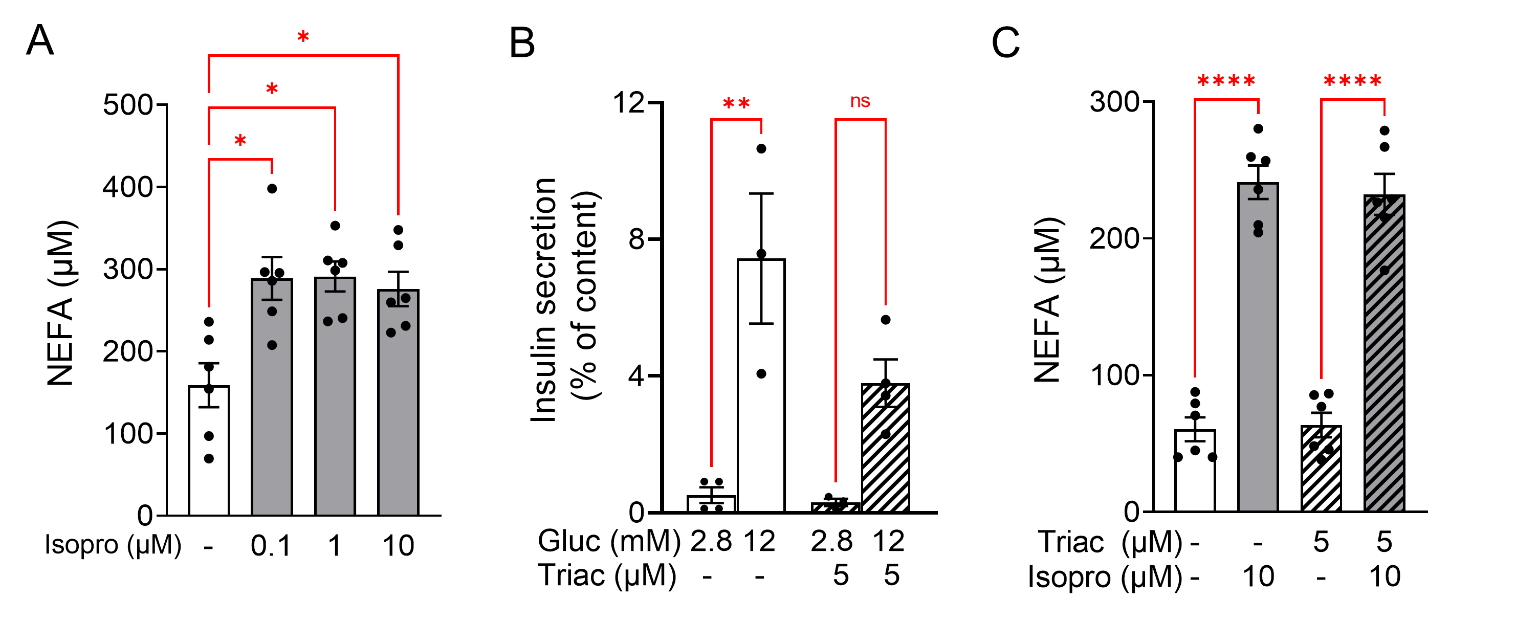


**Suppl. Fig. 3: Effects of isoproterenol and triacsin on NEFA release and insulin secretion.** (A,C) Lipolysis and (B) insulin secretion were conducted as described under Materials and methods. (A) Isoproterenol (Isopro) at lower concentrations maximally stimulated lipolysis of eWAT. (B) Triacsin C (Triac) impairs GSIS. (C) Omission of tracsin C did not affect the lipolytic capacity and quantification of NEFA in eWAT. Data are expressed as means ± SEM of n=3-6 observations.

**References**

1 American Diabetes, A. 2. Classification and Diagnosis of Diabetes: Standards of Medical Care in Diabetes-2021. *Diabetes Care* **44**, S15-S33, doi:10.2337/dc21-S002 (2021).

2 Levy, J. C., Matthews, D. R. & Hermans, M. P. Correct homeostasis model assessment (HOMA) evaluation uses the computer program. *Diabetes care* **21**, 2191-2192 (1998).

3 Siegel-Axel, D. I. *et al.* Fetuin-A influences vascular cell growth and production of proinflammatory and angiogenic proteins by human perivascular fat cells. *Diabetologia* **57**, 1057-1066, doi:10.1007/s00125-014-3177-0 (2014).
